# Ten Recommendations for the Next Clinical Trial of the Mediterranean Diet in Inflamm-Aging: Results & Insights from a Scoping Review

**DOI:** 10.14283/jarlife.2024.18

**Published:** 2024-12-12

**Authors:** A.M.R. Hanna, A.-M.E. Hartford, S. Morassaei

**Affiliations:** 1. Aging & Health Program, Department of Rehabilitation Science, Queen’s University, Kingston, ON, Canada

**Keywords:** Mediterranean diet, clinical trial design, scoping review, inflammation, inflamm-aging, nutrition, molecular

## Abstract

Diet is a key modifiable risk factor in many chronic diseases, including age-related diseases. The Mediterranean diet (MedDiet) is an extensively studied dietary pattern which has been proposed as a lifestyle intervention to promote healthy aging in the general population, due to its numerous health benefits. Randomized controlled trials (RCTs) have attempted to explore the mechanism(s) by which the MedDiet exerts its beneficial effects on aging. One proposed mechanism is that the MedDiet helps to slow down a process called ‘inflamm-aging’, a type of chronic, low-grade inflammation which contributes to aging. To explore the evidence supporting this hypothesized mechanism, we conducted a scoping review of existing RCTs which used a MedDiet intervention and assessed at least one molecular outcome of potential relevance to inflamm-aging. We identified 14 papers representing 12 unique RCTs. Based on our findings, we present 10 recommendations for the next clinical trial of the MedDiet in inflamm-aging.

## Part One: Executive Summary

Diet is a modifiable risk factor for many health conditions, including age-related diseases (ARDs) (1, 2), such as atherosclerosis, cardiovascular disease, certain cancers, type 2 diabetes, and hypertension. As such, healthcare professionals are interested in identifying healthy dietary patterns which can be promoted to the general population.

The Mediterranean Diet (MedDiet) is one of the most extensively studied diet patterns in the world. This centuries-old diet derives its name from the Mediterranean region where it is traditionally consumed (3). While exact definitions vary, it is generally characterized as involving a high intake of unrefined cereals (such as pasta and bread), fruits, vegetables, legumes, nuts, and extra virgin olive oil; moderate intake of poultry, dairy products (mostly light cheeses and yogurts), and alcohol (particularly, red wine); and low intake of red meat, sweets, and processed foods (3-5). Overall, the MedDiet is characterized by a low-glycemic index and an emphasis on plant-based sources of protein (6).

The MedDiet is being studied for its potential role in healthy aging. In fact, several benefits of the MedDiet in promoting healthy aging have been demonstrated in existing studies (5). For instance, adherence to the MedDiet has been shown to offer the following health benefits in regards to age-related decline: it may help in the prevention of muscle mass reduction, mineral bone density reduction, cognitive decline, immune system dysregulation, and cardiovascular diseases (4). Additionally, it can aid in preserving sexual capacity (4), reducing the onset of frailty (5), and increasing the lifespan (5). It seems that adherence to the MedDiet can confer these significant health benefits in aging if adopted by midlife (5, 7).

It would be of interest to understand why the MedDiet confers beneficial health effects to aging. Knowing this could provide new insights into developing new healthy diet interventions or optimizing existing ones. For instance, certain characteristics of the MedDiet, such as its high content of fibre, unsaturated fatty acids, antioxidants, vitamins, and phytochemicals, have been identified as specific components conferring health benefits (8). However, the exact mechanism(s) by which the MedDiet exerts its beneficial effects is unknown (9). Tosti et al. identified potential mechanisms, one of which is protection against inflammation, oxidative stress, and platelet aggregation (9). There is some evidence to support this suggestion; for instance, certain phytochemicals in the MedDiet are known to have anti-inflammatory effects (6, 8, 9). Also, the MedDiet has been shown to beneficially alter composition of the gut microbiome by favouring bacterial strains with anti-inflammatory properties (3).

Researchers are interested in the role of inflammation in aging. Specifically, chronic, sterile, low-grade inflammation is thought to accelerate the aging process through multiple mechanisms (4). It is proposed that this phenomenon, known as ‘inflamm-aging’, occurs due to an excessive immune response to normal stressors (10) due to immune system dysregulation with age (11). This contributes to the progression of ARDs (12) such as cardiovascular diseases, type 2 diabetes, cognitive decline, dementia, frailty, sarcopenia, and cancer (5). Relevant molecules in inflamm-aging include the transcription factor Nuclear Factor kappa B (NFκB) (12); inflammatory cytokines (12) such as interleukins, tumour necrosis factor alpha (TNFα), and interferon gamma (IFNγ); and C-reactive protein (CRP) (13).

Knowing that the MedDiet appears to have beneficial health effects regarding to aging and ARDs, and considering the emerging evidence of its anti-inflammatory effects, we decided to conduct a scoping review on the current state of evidence regarding the role of the MedDiet in reducing inflammation at the molecular level. We asked the question: “How is consumption of a MedDiet related to molecular changes of potential relevance to inflamm-aging?”

To conduct this scoping review, we searched four databases from inception to January 10, 2023, looking for articles that referred both to a MedDiet and to the inflammatory process at the molecular level. We included any randomized clinical trial (RCT) in healthy adult patients who were randomized to receive either a MedDiet or a control diet, and in which at least one molecular outcome related to the inflammatory process was measured. For instance, the study could look at the amount of transcription or translation of a protein involved in inflammation, such as a cytokine. Specifically, that molecule should be involved in a chronic, sterile inflammation i.e., inflamm-aging, regardless of whether the article specifically referred to the process as “inflamm-aging”. We decided to focus on RCTs which included a general population of adults, rather than a subset with a specific diagnosis, as the ultimate goal is to inform policy on whether or not the MedDiet is a useful lifestyle intervention for healthy aging for adults in general. Thus, we excluded RCTs for which a medical diagnosis, among other criteria, were required for enrolment. In all, we screened 4,614 articles and included 14 articles representing 12 RCTs. The included studies are summarized in Table 1. The inclusion and exclusion criteria are detailed in Table 2.

**Table 1:** Summary of Included Studies

Article	Total sample size (n)	Arm length	Baseline diet	Socioeconomic status (SES) & Race/ Ethnicity	Intervention and control diets	Caloric state	Molecular changes assessed	Results (MedDiet vs. Control**)
1. Ambring et al., 2006 (18)	22	4 wk	NR	NR	Med-inspired diet vs. Ordinary Swedish diet	NR	Serum level of hs-CRP, MCP-1, IL-6	NS for all
2. Camargo et al., 2012 (19)	20	4 wk	NR	NR	MedDiet + VOO vs. SFA diet vs. CHO-PUFA diet	NR	Activation of NFkB; expression of p65, IκBα, MCP-1, TNFα, IL-6, MIF-1, MMP-9	↓ p65, MCP-1, MMP-9 All others NS
3. Clements et al., 2017 (21)	120	1 yr	NR	NR	MedDiet vs. HabDiet	NR	Production of MCP-1, CXCL8, resistin, TNFα by isolated PBMCs	NS for all
4. Davis et al., 2017 (16)	166	6 mo	NR	NR	Australianized MedDiet vs. HabDiet	Iso caloric	Serum level of hs-CRP	NS
5. Djuric et al., 2009 (24)	69	6 mo	Inclusion criteria	SES: Reported % college graduates Race / Ethnicity: Reported % White	Modified MedDiet vs. HabDiet	NR	Serum level of CRP	NS
6. Jaacks et al., 2018 (26)	20^*^	8 wk	Inclusion criteria and reported actual baseline diet	NR	MedDiet vs. high fat American-type HabDiet	NR	IL-6, IL-8, CRP, adiponectin	NS for all
7. Konstantinidou et al., 2010 (23)	90	3 mo	Inclusion criteria	NR	MedDiet + WOO vs. MedDiet + VOO vs. HabDiet	NR	Serum level IFNγ, MCP-1, s-Ps, S-CD40L, hs-CRP; Expression of ARHGAP15, IFNγ, IL-7R	↓ ARHGAP15, IFNγ All others NS
8. Lopez-Moreno et al., 2018 (14)	20	4 wk	Inclusion criteria	NR	MedDiet + CoQ vs. MedDiet + placebo vs. Western SFA diet	Iso caloric	Serum level of dAGE; mRNA level of MG, CML, AGER1, RAGE, Gloxl, ERa	↓ dAGE ↓ MG, CML, RAGE ↑ AGER1, Gloxl, ERa
9. Maijo et al., 2018 (22)	122	1 yr	NR	NR	MedDiet + Vit D vs. HabDiet	NR	Production of IFNα, IFNβ, IFNγ, IL-12p40, IL-12p70, IL-12rβi, IL-12rβ2, SOCS3 by isolated PBMCs	NS for all
10. Meslier et al., 2020 (27)	82	8 wk	Run-in diet and inclusion criteria	NR	MedDiet vs. HabDiet	NR	Serum level of hs-CRP	NS
11. Perez-Martinez et al., 2007 (20)	16	4 wk	NR	NR	MedDiet + VOO vs. Western SFA Diet vs. High-CHO + vegetal n-3 FAs Diet	NR	Activation of NFκB in PBMCs Serum level of VCAM-1, ICAM-1, MCP-1, IL-6, TNFα	↓ NFκB (activation) ↓ VCAM-1 All others NS
12. Stendell-Hollis et al., 2013 (25)	129	4 mo	Run-in and washout diets	SES: Reported % college graduates Race / ethnicity: Reported % White and Hispanic	Med-style Diet vs. MyPyramid Diet	NR	Serum level of IL-6, TNFα	NS for all
13. van Dijk et al., 2012 (28)	60	8 wk	Run-in diet	NR	MedDiet vs. Western-type SFA diet vs. Western-type MUFA diet	NR	Serum level of IL-1β, Factor VII, MIP-lα, SAP, TP, VEGF, IL-12p70, CRP, IL-10, IL-11, IL-13, IL-15, IL-16, IL-17, IL-18, IL-23, IL-3, IL-4, IL-5, IL-6, IL-7, IL-8, IL-1RA, MCP-1, MCSF, MDC, MIP-1β, TNF α-RTIII, VCAM-1	NS for all
14. Yubero-Serrano et al., 2012 (15)	20	4 wk	Inclusion criteria	NR	MedDiet + CoQ vs. MedDiet + placebo vs. Western SFA diet	Iso caloric	mRNA level of p65, IKKβ, IκBα, MMP9, IL-1β, JNK-1	↓ p65, IKKβ, MMP9, IL-1β, JNK-1 ↑ IκBα

*Note: The actual total sample size for the Jaacks et al., 2018 study is n=27 because there is a second intervention arm (“Supplements diet”). This is NR for the present scoping review as it was not treated as a control compared to the MedDiet; therefore, the results of this diet do not help to answer the research question. **Note: Some studies compared more than one MedDiet to more than one control. For those interested in the finer details beyond what is presented in the executive summary, please see Appendix 2B. NR: Not reported; CHO: Carbohydrate; CoQ: Coenzyme Q; EVOO: Extra virgin olive oil; HabDiet: Habitual diet; MUFA: Monounsaturated fatty acid; PUFA: Polyunsaturated fatty acid; RDA: Recommended daily allowance; SFA: Saturated fatty acid; USDA: United States Department of Agriculture; VOO: Virgin olive oil; WOO: Washed olive oil; PBMCs: Peripheral blood mononuclear cells; For full names of molecule acronyms, please see Appendix 2F.

**Table 2:** Inclusion & Exclusion Criteria

TITLE & ABSTRACT SCREENING	
Inclusion Criteria	Exclusion Criteria
Study Design: Experimental study such as RCT, comparing MedDiet to another type of diet; AND Article Type: Original Research; AND Population: Adult humans (18+ years old) of any health status; AND Experimental intervention: Described by authors as a Mediterranean diet (MedDiet), MedDiet supplemented with additional component(s) (e.g., MedDiet + EVOO), or ‘Med-style/type/like’ diet; AND Outcome: Assessed change(s) in any measurable aspect of one or more biological molecules; AND Any mention of inflammation, inflamm-aging, the inflammatory response, the immune system, AND/OR mention of at least one of the following molecules: cytokines, chemokines, interleukins, interferons, lymphokines, monokines, TGFβ, TNFa, CRP, or NFκB.	Observational studies whose conclusions are only correlational, such as cohort studies, case-control studies, cross-sectional studies (including surveys/questionnaires), and quasi-experiments; AND/OR Studies conducted in vitro, with animals, or children (<18 years old); AND/OR Experimental intervention is a single component of the MedDiet, such as olive oil, nuts, herbs, or phytochemicals, or a macronutrient such as MUFAs, as opposed to a full MedDiet; AND/OR Experimental intervention is a lifestyle intervention that includes MedDiet but also involves (a) non-diet intervention(s) such as physical activity; AND/OR Experimental intervention is only counselling in MedDiet principles; AND/OR Articles not reporting on the results of an experiment, such as review articles, opinion pieces, case studies, or methodologies; AND/OR Dissertations, reports, and conference abstracts.
**FULL-TEXT REVIEW**	
**Inclusion Criteria**	**Exclusion Criteria**
Study Design: RCT (with or without crossover); AND Study Design: At least one statistical comparison must be MedDiet vs. non-MedDiet; AND Article Type: Original Research; AND Population: Adult humans (18+ years old) Generalizable population – defined as enrolment in the trial not requiring current medical diagnosis (e.g., cancer, obesity) NOR current elevated risk of disease (e.g., moderate/high CVD risk - whether assessed by doctor or research team - Lifestyle risk factors OK) NOR currently abnormal test results (e.g., hypercholesterolemia) NOR post-operative; AND Experimental intervention: Described by authors as a Mediterranean diet (MedDiet), MedDiet supplemented with additional component(s) (e.g., MedDiet + EVOO), or ‘Med-style/type/like/etc’ diet (can be hypo/iso/hypercaloric); AND Outcome: Assessed change(s) in any measurable aspect of one or more biological molecules in the participants; AND At least one of the assessed molecules is explicitly stated by authors as being implicated in inflamm-aging or inflammation AND/OR is one of the following molecules (known to be involved in inflamm-aging/inflammation): cytokines, chemokines, interleukins, interferons, lymphokines, monokines, TGFβ, TNFa, CRP, or NFκB. Language: English.	Observational studies whose conclusions are only correlational, such as cohort studies, case-control studies, cross-sectional studies (including surveys/questionnaires), and quasi-experiments; AND/OR Studies conducted in vitro^1^, with animals, or children (<18 years old); AND/OR Experimental intervention is a single component of the MedDiet, such as olive oil, nuts, herbs, or phytochemicals, or a macronutrient such as MUFAs, as opposed to a full MedDiet; AND/OR Experimental intervention is described by authors as a combination MedDiet + another diet pattern^2^ (e.g., Med-Keto diet); AND/OR Intervention is a single meal (i.e., MedMeal; e.g., gazpacho soup); AND/OR Experimental intervention is a lifestyle intervention that involves MedDiet but also includes non-diet component(s) such as physical activity; AND/OR Experimental intervention is only counselling in general MedDiet principles without any structured effort at adherence (examples of «structured effort» include but are not limited to at least one of: clear instructions as to allowed food/macronutrient proportions, post-intervention adherence questionnaire, follow-up phone call); AND/OR Article describes the effect of gene variants (polymorphisms) on response to MedDiet, rather than MedDiet vs. non-MedDiet comparison; AND/OR Articles not reporting on the results of an experiment, such as review articles, opinion pieces, case studies, or methodologies; AND/OR Publications that are not full original articles such as dissertations, reports, poster presentations, and conference abstracts; AND/OR Assessed molecules are described by authors as being exclusively involved in inflammation that is not characteristic of inflamm-aging i.e., acute, non-sterile (in response to infection), and/or high-grade inflammation.

1. An experiment performed in vitro is acceptable if the sample originated from study participant; 2. Adjustments of MedDiet to local cultural or other preferences are acceptable.

Based on the results of our scoping review, we have developed the following list of 10 recommendations for designing a future clinical trial of the MedDiet in inflamm-aging.

**1. Consider larger sample sizes to detect smaller effect sizes.** The included studies varied in sample size from 20 (14, 15) to 166 participants (16). The sample size necessary for an adequately powered RCT looking at molecular changes relevant to inflamm-aging is most likely much higher. For instance, if we consider a continuous variable such as high sensitivity CRP (hs-CRP), if we would like to detect changes of at least 0.1 mg/dL (normal range is <0.3 mg/dL, with 0.3-1.0 mg/dL being considered “minor elevation” (17)), the estimated overall samples size for a two-arm study in which there is a 10 or 15% dropout rate are 872 to 922, respectively (if interested, please see Part Three: Detailed Methods and Results for the full calculation).

**2. Collect data at multiple timepoints, for at least 1 year.** The duration of time participants received a diet intervention or control varied from 4 weeks (14, 15, 18-20) to 1 year (21, 22). Most (n=5/9) short-term studies (4 weeks to 3 months) had statistically significant results (14, 15, 19, 20, 23), whereas most (n=4/5) long-term studies (4 months to 1 year) had statistically insignificant results (16, 22, 24, 25), regardless of the molecules, genes, or types of changes assessed. As we found that most significant results were observed in short-term studies, the MedDiet may have a transient effect on inflammation. Future studies should be long term (e.g., 1 year) and repeat measurements at multiple timepoints (e.g., 1, 3, 6, 9, 12 months), to evaluate the effect of the MedDiet over time.

**3. Collect information on baseline diet.** Only five studies had inclusion criteria for baseline diet (14, 15, 23, 24, 26, 27) and only three studies had a run-in or washout diet prior to randomization (25, 27, 28). Only one study reported actual baseline diet of participants (26). This is a potential confounding factor, as we cannot know whether the intervention and control groups had similar baseline diets. This also makes it difficult to compare between RCTs.

**4. Collect information on patient socioeconomic status (SES).** All three components of SES (occupation, education, and income level) are related to quality of diet (29). For instance, lower SES individuals tend to have higher intake of white bread and refined cereals, whereas higher SES individuals tend to consume more wholegrain products (29). Also, higher SES individuals tend to consume greater quantities of fruit and vegetables than lower and middle SES individuals (29). As the MedDiet places an emphasis on fruits, vegetables, and wholegrain, unrefined cereals, we would expect studies with a higher SES population to have a baseline diet more closely resembling the MedDiet. Despite this, only two studies reported on SES, of which the only factor considered was educational attainment, not income or occupation (24, 25). Furthermore, educational attainment information was incomplete for these studies, as they reported only on percentage of college graduates without providing the remaining breakdown (24, 25).

**5. Collect information on race/ethnicity and include diverse populations.** Similarly, only two studies reported race/ethnicity (the same two which reported on SES) (24, 25). Race/ethnicity could have implications for dietary studies (30) which could influence both participant baseline diet (especially for studies with HabDiet controls) and participant response to a MedDiet intervention. Including a diverse population in future RCTs would increase generalizability.

**6. All study participants should follow an isocaloric diet, regardless of allocation.** Most (n=11) studies did not report the caloric state of their experimental and control arms. The Western diet, which is calorically rich, has been shown to create a state of chronic inflammation (31). Conversely, caloric restriction has been shown to reduce systemic inflammation (11). Therefore, if studies had different caloric states between arms, this could create a confounding variable which could affect participant inflammatory status. Future studies should track caloric intake and compare it to expenditure to ensure that all trial arms have the same caloric excess or deficit. An isocaloric diet avoids this confounding and is an important control for both the intervention and control diets. The caloric intake of participants should be tracked over time to ensure it is both (i) isocaloric and (ii) comparable between arms.

**7. Use a MedDiet intervention defined by flexible dietary patterns, not fixed macronutrient proportions, and assess adherence.** Studies reported variety in the types of MedDiets used. The most common were self-described regular/traditional MedDiets (n=3) (18, 26-28) and MedDiet + virgin olive oil (VOO) (n=3) (19, 20, 23). Two RCTs used a MedDiet plus additional component, being coenzyme Q (CoQ) in one RCT (14, 15) and vitamin D in another (22). The remaining four articles described their intervention as some variation of the MedDiet such as a Med-inspired diet (n=1) (18), Med-style diet (n=1) (25), or modified MedDiet (n=2) (16, 24). At the surface, the most academically satisfying RCT might appear to be a precisely defined MedDiet with percentage macronutrient distributions (e.g., “5% of calories from monounsaturated fatty acids”), but on reflection this is unlikely to be a sustainable approach that most individuals would practice over the long term – they may not have the time, desire, knowledge, or resources to do so. Our ultimate goal should be to create a clinically relevant RCT that answers whether the intervention as it would be administered by a healthcare provider (e.g., a primary care provider recommending that a patient follow a MedDiet) is relevant to inflamm-aging. Therefore, we recommend that the RCT present the MedDiet intervention as a flexible dietary pattern; for instance, through one or both of the methods suggested by the Fundación Dieta Mediterránea (32): (i) basic recommendations (e.g., “eat plenty of fruits and vegetables”) and/or (ii) specific serving targets in the MedDiet pyramid (e.g., “1-2 servings of fruits per day, at least 2 servings of vegetables per day”). This would also allow people to more easily modify the MedDiet to suit their tastes and cultural background. Additionally, a MedDiet adherence score such as the 18-point score proposed by Sofi et al. (33) could be used to assess adherence and ensure it sufficiently differs from the control diet. Finally, it would be interesting to assess participant satisfaction with the MedDiet intervention and compare that to the control diet, to predict likelihood of long-term adherence. Ultimately, a healthcare provider can counsel a patient to adopt a MedDiet, but if the patient cannot implement it (e.g., it is too time-consuming, expensive, or complicated), then the intervention will not contribute to improving that patient’s health, and we would be performing an RCT which may be more focused on academic interests than pragmatic objectives.

**8. Use a Habitual Diet (HabDiet) control, know what participants’ HabDiet is, and know if it is sufficiently different from MedDiet.** The most common control diet was the Habitual diet (HabDiet), which was used in seven studies (16, 21-24, 26, 27). Of the seven studies which used HabDiet controls, only one reported baseline participant diet (26). Three other articles had inclusion criteria regarding participant baseline diet but did not report actual diet (23, 24, 27). Finally, another three articles had neither (16, 21, 22). While half (n=7) of studies used a HabDiet control, only one reported baseline participant diet (26). As mentioned earlier, this is an important limitation, since it means we have limited information as to what diet the intervention group was being compared to. If the HabDiet of participants in some studies already resembled a MedDiet, the comparison between two very similar diets could explain the non-significant results. Future studies using a HabDiet should have inclusion criteria regarding HabDiet characteristics, to ensure that any participants randomized to the HabDiet have a sufficiently different baseline diet from the MedDiet intervention to allow for meaningful comparison. As well, HabDiet participants should be required to complete a food diary.

**9. Use caution regarding a Western diet control.** The second most common control diet was the Western/SFA (Saturated Fatty Acid) diet which was used in four studies (14, 15, 20, 28). It is possible that most study participants’ HabDiet will already be a Western diet (that will be determined by food diaries). However, we do not recommend that RCTs randomize participants to a Western diet, for two reasons. Firstly, there is probably not clinical equipoise regarding the Western diet, which is known to be an unhealthy pro-inflammatory diet (34) which increases the likelihood of obesity (35). Not being in a state of clinical equipoise regarding the interventions being tested is one of the ‘transgressions of trialists’ identified by Meinert (36). Secondly, if participants’ HabDiet does not match a Western diet, especially depending on how that diet is defined, then the control diet could be considered an ‘exaggerated’ diet which may not be representative of the HabDiet of the average patient seeing their healthcare provider in that region. Again, our goal should be to create the most clinically relevant RCT possible.

**10. Assess a wide variety of molecular changes, both upstream and downstream in inflammation.** A total of 91 molecular changes were assessed across all 14 studies, with only 20 being statistically significant between MedDiet and control across six articles (14, 15, 19-21, 23). Overall, a total of 59 unique molecules and genes were assessed. The most common molecular change assessed was serum levels of molecules, in 11 studies (14, 16, 18-20, 23-28). Other common changes assessed included gene expression (19, 23), production by peripheral blood mononuclear cells (PBMCs) (21, 22), and mRNA levels (14, 15). The most commonly assessed molecules were cytokines (such as interleukins, tumour necrosis factor alpha (TNFα), and interferons) in nine studies (18-23, 25, 26, 28); CRP and hs-CRP collectively in six studies (16, 18, 23, 24, 27, 28); and nuclear factor kappa B (NFκB) and its associated molecules (i.e., p65 subunit, IKKβ, IκBα) in three studies (15, 19, 20). Despite the anti-inflammatory effect of the MedDiet on NFκB at multiple levels and for its related molecules, studies did not show significant effects for cytokines (15, 18-20, 23, 25, 26, 28) nor CRP and hs-CRP (16, 18, 23, 24, 26, 27). While consumption of a MedDiet may create molecular changes of potential relevance to inflamm-aging, based on existing studies these appear to be mostly limited to the NFκB signalling pathway. Interestingly, even though NFκB regulates production of pro-inflammatory cytokines (37), this did not correspond to significant changes in cytokine production. Furthermore, lower CRP or hs-CRP would be expected in individuals with lower inflamm-aging, as these molecules are among its key mediators (13), but this was not observed. Overall, this suggests that while a MedDiet creates a statistically significant reduction in NFκB signalling, this reduction may not be of a sufficient effect size to create a meaningful downstream reduction in pro-inflammatory cytokines or CRP and hs-CRP, based on existing studies. Thus, we recommend that future studies look at a wide variety of molecular changes, including NFκB and its associated molecules, a panel of inflammatory cytokines, and CRP or hs-CRP.

## Part Two: Consolidated Methods and Results

We searched four databases (MEDLINE, Web of Science, EMBASE, CINAHL) for relevant articles on the MedDiet and inflamm-aging. Searches were conducted on January 10, 2023, and included articles in English since database inception.

Title and abstract and full-text screening were conducted by two reviewers (AMRH & AMEH) using the inclusion and exclusion criteria presented in Table 2. Key data extracted include individual study characteristics (including location, setting, and duration) baseline population characteristics (including age, sex, baseline diet, chronic disease, and social factors), dietary intervention details (description of experimental and control arms), and molecular changes (qualitative assessment of directional change for MedDiet intervention vs. control). Quality assessment was conducted using the Critical Appraisal Skills Programme (CASP) Randomized Controlled Trial (RCT) checklist, 2020 version. Article screening, data extraction, and quality assessment were conducted by two reviewers (AMRH & AMEH). Conflicts were resolved by consensus.

In total, 14 articles were included, representing 12 unique RCTs. Of these 12 RCTs, 4 were crossover studies (14, 15, 18-20). The RCTs were conducted in seven different countries, the majority (n=10) of which were in Europe. Three RCTs were in the USA (24-26) and one in Australia (16). All articles except one (24) reported on single-centre studies. Two articles (21, 22) reported on subsets of a larger multi-centre trial. Sample sizes ranges from 20 (14, 15) to 166 participants (16).

As per the inclusion criteria, we only included studies with adults. The mean age ranged from 29.7 years (25) to 71.0 years (16). Three articles did not report mean age (14, 15, 20). Of the 10 studies which reported mean body mass index (BMI), six included participants with a mean BMI considered overweight (15, 16, 18, 21, 22, 25). Most studies included both males and females, except two studies which included only females (24, 25) and one study which included only males (20).

Only five studies had inclusion criteria for baseline diet (14, 15, 23, 24, 26, 27) and only three studies had a run-in or washout diet prior to randomization (25, 27, 28). Only one study reported actual baseline diet of participants (26).

Only two studies reported on SES and race/ethnicity (24, 25). For SES, only education was listed (not income nor occupation). Educational attainment information was incomplete for both studies, reporting only on percentage of college graduates without providing the remaining breakdown (24, 25). Of these two studies, one provided only a partial breakdown of race/ethnicity with only the proportion of white participants reported (24).

Studies reported variety in the types of MedDiets used. The most common were self-described regular/traditional MedDiets (n=3) (18, 26-28) and MedDiet + virgin olive oil (VOO) (n=3) (19, 20, 23). Two RCTs used a MedDiet plus additional component, being coenzyme Q (CoQ) in one RCT (14, 15) and vitamin D in another (22). The remaining four articles described their intervention as some variation of the MedDiet such as a Med-inspired diet (n=1) (18), Med-style diet (n=1) (25), or modified MedDiet (n=2) (16, 24).

Half (n=7) of articles provided a breakdown of the proportion of macronutrients (protein, carbohydrates, and fat) as percent daily energy (14-16, 18-20, 22). The other half (n=7) provided either only a macronutrient partial breakdown (n=2) (26, 28), serving requirements/recommendations (n=3) (21, 25, 27), a combination of serving requirements/recommendations and macronutrient ratios (n=1) (24), or individualized advice to increase MedDiet score (n=1) (23).

The most common control diet was the Habitual diet (HabDiet), which was used in seven studies (16, 21-24, 26, 27), followed by a Western/SFA (Saturated Fatty Acid) diet which was used in four studies (14, 15, 20, 28). Of the seven studies which used HabDiet controls, only one reported baseline participant diet (26). Three other articles had inclusion criteria regarding participant baseline diet but did not report actual diet (23, 24, 27). Finally, another three articles had neither (16, 21, 22). Only three studies reported on the caloric state of their intervention and control arms (14-16).

A total of 91 molecular changes were assessed across all 14 studies, with only 20 being statistically significant between MedDiet and control across six articles (14, 15, 19-21, 23). Of those 20 changes, all were consistently anti-inflammatory (14, 15, 19-21, 23), meaning that a molecule with a pro-inflammatory role significantly decreased in the MedDiet group versus control, or significantly increased for a molecule with an anti-inflammatory role.

Overall, a total of 59 unique molecules and genes were assessed. The most common molecular change assessed was serum levels of molecules, in 11 studies (14, 16, 18-20, 23-28). Other common changes assessed included gene expression (19, 23), production by peripheral blood mononuclear cells (PBMCs) (21, 22), and mRNA levels (14, 15).

The most commonly assessed molecules were cytokines (such as interleukins, tumour necrosis factor alpha (TNFα), and interferons) in nine studies (18-23, 25, 26, 28); C-reactive protein (CRP) and high sensitivity CRP (hs-CRP) collectively in six studies (16, 18, 23, 24, 27, 28); and nuclear factor kappa B (NFκB) and its associated molecules (i.e., p65 subunit, IKKβ, IκBα) in three studies (15, 19, 20).

Some studies showed a significant effect on NFκB, in terms of its p65 subunit expression (19), its activation in PBMCs (20), and its mRNA levels and mRNA levels of related molecules in the signalling pathway (i.e., IKKβ and IκBα) (15). However, this effect was not consistently observed across all studies, and in addition, one study found a non-significant effect of the MedDiet on expression of NFκB and IκBα compared to two control diets (19). Despite the anti-inflammatory effect of the MedDiet on NFκB at multiple levels and for its related molecules, studies did not show significant effects for cytokines (15, 18-20, 23, 25, 26, 28) nor CRP and hs-CRP (16, 18, 23, 24, 26, 27).

Most (n=5/9) short-term studies (4 weeks to 3 months) had statistically significant results (14, 15, 19, 20, 23), whereas most (n=4/5) long-term studies (4 months to 1 year) had statistically insignificant results (16, 22, 24, 25), regardless of the molecules, genes, or types of changes assessed.

## Part Three: Detailed Methods and Results

### Detailed methods

#### Search strategy

We searched four databases (MEDLINE, Web of Science, EMBASE, CINAHL) for relevant articles focused on the MedDiet and inflamm-aging (see Appendix 1A for themes and keywords). The searches were conducted on January 10, 2023, and included all articles in English since database inception. Appendices 1B-E present the complete search strategies for each database. This review was registered with Open Science Framework: https://osf.io/cpgqw.

#### Screening & selection process

**Figure 1:**
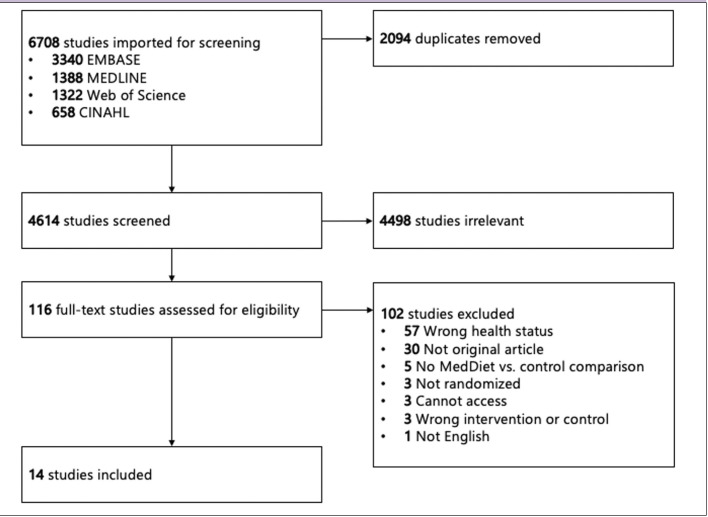
PRISMA Diagram

Title and abstract and full-text screening were conducted by two reviewers (AMRH & AMEH) using Covidence systematic review software (Veritas Health Innovation, Melbourne, Australia) using the inclusion and exclusion criteria as presented in Table 2. Duplicates were removed after importation of studies into Covidence. Conflicts were resolved by consensus. The PRISMA diagram is shown in Figure 1.

#### Data extraction

Data extraction was conducted by two reviewers (AMRH & AMEH) using Microsoft Excel (Microsoft Corporation, Redmond, US). Conflicts were resolved by consensus.

Key data extracted include individual study characteristics (including location, setting, and duration) baseline population characteristics (including age, sex, baseline diet, chronic disease, and social factors), dietary intervention details (description of experimental and control arms), and molecular changes (qualitative assessment of directional change for MedDiet intervention vs. control). Tables containing all extracted data can be found in Appendix 2.

#### Quality assessment

Quality assessment was conducted by two reviewers (AMRH & AMEH) using the Critical Appraisal Skills Programme (CASP) Randomized Controlled Trial (RCT) checklist, 2020 version. Conflicts were resolved by consensus. The quality assessment table is presented in Appendix 2E.

Scoring was conducted using the following principles, adapted from Arantzamendi et al. (38): Yes (Y) = 1 point, No (N) or Can’t tell (C) = 0 points. For question 4, which is split into three sub-questions, each Y = 1/3 point. Therefore, each study will receive a score out of 11. A score of 5 or less was considered “low” quality, 5 1/3 to 8 2/3 was considered “moderate” quality, and 9 or higher was considered “high” quality.

#### Synthesis

AMRH synthesized articles using the simplified thematic analysis approach outlined by Aveyard (39). To generate a theme, at least three articles were required, at least one of which had to be a high-quality study.

### Limitations of methods

First, as it is not feasible to make a list of every single molecule potentially involved in inflamm-aging or inflammation, articles had to refer to one of the main molecules known to be involved in inflamm-aging or inflammation (see Table 2), or the molecule had to be explicitly stated as involved in inflamm-aging or inflammation to be included. Therefore, we cannot rule out the possibility that we have missed articles reporting on relevant molecules which were neither in this list nor explicitly stated by the authors as involved in inflamm-aging or inflammation.

Second, the search strategies (see Appendices 1B-E) exploded subject headings where possible, but not every minor subheading was included as a keyword for redundancy. For example, we included both the subject heading “Monokines” (under exploded “Cytokines”) and the keyword “monokine*” in MEDLINE but not the keyword “Oncostatin M”, which appears under the “Monokines” subject heading tree. Therefore, we may have missed articles reporting on a molecule such as Oncostatin M, if no other search terms appeared in the title or abstract.

Third, we did not include all alternate names of molecules as search terms. For example, while we included the exploded subject heading “Interleukins” (under exploded “Cytokines”) and the keyword “interleukin*” in MEDLINE, we did not include as keywords terms such as “epidermal cell derived thymocyte activating factor”, an alternate name for interleukin 1.

Fourth, we limited our search and selection to articles in English. It is possible that relevant articles in other languages were not included.

### Detailed results: Descriptive overview

#### Study characteristics

In total, 14 articles were included, representing 12 unique RCTs. Of these 12 RCTs, 4 were crossover studies (14, 15, 18-20). The RCTs were conducted in seven different countries, the majority (n=10) of which were in Europe, of which nearly half (n=4) were conducted in Spain (14, 15, 19, 20, 23). Only four studies were conducted outside of Europe: three in the USA (24-26) and one in Australia (16).

All articles except one (24) reported on single-centre studies. Two articles (21, 22) reported on subsets of a larger multi-centre trial. Sample sizes ranged from 20 (14, 15) to 166 participants (16). Please see Appendix 2A for full details.

#### Baseline population characteristics

As per the inclusion criteria, we only included studies with adults. The mean age ranged from 29.7 years (25) to 71.0 years (16). Three articles did not report mean age (14, 15, 20). Of the 10 studies which reported mean body mass index (BMI), six included participants with a mean BMI considered overweight (15, 16, 18, 21, 22, 25). Most studies included both males and females, except two studies which included only females (24, 25) and one study which included only males (20).

All but two studies included only individuals without clinically diagnosed chronic diseases. One study had a total of 11 cases of chronic disease despite describing their study population (n=20 participants) as “healthy” at baseline (19). Another study had a sample exclusion criterion of a lengthy but not necessarily comprehensive list of chronic diseases (23).

Most studies included samples drawn from the general population; however, one study included only medical students (20), and another included only postpartum breastfeeding women (25).

Only five studies had inclusion criteria for baseline diet (14, 15, 23, 24, 26, 27) and only three studies had a run-in or washout diet prior to randomization (25, 27, 28). Only one study reported actual baseline diet of participants (26).

Only two studies reported on SES and race/ethnicity (24, 25). For SES, only education was listed (not income nor occupation). Educational attainment information was incomplete for both studies, reporting only on percentage of college graduates without providing the remaining breakdown (24, 25). Of these two studies, one provided only a partial breakdown of race/ethnicity with only the proportion of white participants reported (24). Please see Appendix 2C for full details.

#### Dietary intervention characteristics

Studies reported variety in the types of MedDiets used. The most common were self-described regular/traditional MedDiets (n=3) (18, 26-28) and MedDiet + virgin olive oil (VOO) (n=3) (19, 20, 23). Two RCTs used a MedDiet plus additional component, being coenzyme Q (CoQ) in one RCT (14, 15) and vitamin D in another (22). The remaining four articles described their intervention as some variation of the MedDiet such as a Med-inspired diet (n=1) (18), Med-style diet (n=1) (25), or modified MedDiet (n=2) (16, 24).

Half (n=7) of articles provided a breakdown of the proportion of macronutrients (protein, carbohydrates, and fat) as percent daily energy (14-16, 18-20, 22). Among these studies, the range was 15% to 20% protein, 47% to 55% carbohydrate, and 38% to ~40.5% fat. The other half (n=7) provided either only a macronutrient partial breakdown (n=2) (26, 28), serving requirements/recommendations (n=3) (21, 25, 27), a combination of serving requirements/recommendations and macronutrient ratios (n=1) (24), or individualized advice to increase MedDiet score (n=1) (23).

The most common control diet was the Habitual diet (HabDiet), which was used in seven studies (16, 21-24, 26, 27), followed by a Western/SFA (Saturated Fatty Acid) diet which was used in four studies (14, 15, 20, 28). Of the seven studies which used HabDiet controls, only one reported baseline participant diet (26). Three other articles had inclusion criteria regarding participant baseline diet but did not report actual diet (23, 24, 27). Finally, another three articles had neither (16, 21, 22).

Only three studies reported on the caloric state of their intervention and control arms (14-16). Only four studies specified the frequency of meals and/or snacks (15, 20, 25, 26). Please see Appendix 2D for full details.

#### Molecular changes

A total of 91 molecular changes were assessed across all 14 studies, with only 20 being statistically significant between MedDiet and control across six articles (14, 15, 19-21, 23). Overall, a total of 59 unique molecules and genes were assessed. The most common molecular change assessed was serum levels of molecules, in 11 studies (14, 16, 18-20, 23-28). Other common changes assessed included gene expression (19, 23), production by peripheral blood mononuclear cells (PBMCs) (21, 22), and mRNA levels (14, 15).

The most commonly assessed molecules were cytokines (such as interleukins, tumour necrosis factor alpha (TNFα), and interferons) in nine studies (18-23, 25, 26, 28); C-reactive protein (CRP) and high sensitivity CRP (hs-CRP) collectively in six studies (16, 18, 23, 24, 27, 28); and nuclear factor kappa B (NFκB) and its associated molecules (i.e., p65 subunit, IKKβ, IκBα) in three studies (15, 19, 20). Please see Appendix 2B for full details.

#### Quality assessment

Half (n=7) of the included articles were considered high quality and half (n=7) were considered moderate quality; none were low quality (Appendix 2E). The lowest score was 6 out of 11 and the highest was 10 2/3 out of 11. Of note, no studies received points for investigator blinding to dietary intervention, as it was either not performed or it was unclear whether it was performed for each study.

### Detailed results: Major themes

#### MedDiet has anti-inflammatory effect in minority of molecules

As previously stated, only 20 of 91 molecular changes were statistically significant across studies. Of those 20 changes, all were consistently anti-inflammatory (14, 15, 19-21, 23), meaning that a molecule with a pro-inflammatory role significantly decreased in the MedDiet group versus control, or significantly increased for a molecule with an anti-inflammatory role.

#### Significant effect on NFκB

Some studies showed a significant effect on NFκB, in terms of its p65 subunit expression (19), its activation in PBMCs (20), and its mRNA levels and mRNA levels of related molecules in the signalling pathway (i.e., IKKβ and IκBα) (15). However, this effect was not consistently observed across all studies, and in addition, one study found a non-significant effect of the MedDiet on expression of NFκB and IκBα compared to two control diets (19).

#### Non-significant effect on cytokines and CRP

Despite the anti-inflammatory effect of the MedDiet on NFκB at multiple levels and for its related molecules, studies did not show significant effects for cytokines (15, 18-20, 23, 25, 26, 28) nor CRP and hs-CRP (16, 18, 23, 24, 26, 27).

#### Longer interventions correlated with non-significant results

Most (n=5/9) short-term studies (4 weeks to 3 months) had statistically significant results (14, 15, 19, 20, 23), whereas most long-term studies (n=4/5) (4 months to 1 year) had statistically insignificant results (16, 22, 24, 25), regardless of the molecules, genes, or types of changes assessed.

### Example sample size calculation

Let us consider an outcome measure such as high sensitivity C-reactive protein (hs-CRP). The normal value for hs-CRP is < 0.3 mg/dL, with values 0.3-1.0 mg/dL being considered “minor elevation”, such as what might be seen in certain chronic diseases such as diabetes or being correlated with risk factors such as obesity or sedentary lifestyle (17).

Using the following equation (modified from Meinert (36)), we can estimate the necessary sample size:


$$n=\frac{2.{\left(Z_{\alpha }+Z_{\beta }\right)}^{2}.\sigma^{2}}{\delta^{2}}$$

Where:

- n = sample size, per arm.
- Z(α/2)= the Z-statistic corresponding to the significance level where α = probability of type I error, for a two-tailed test, i.e., in which we would like to detect changes in hs-CRP in both directions. Usually, α is set to 0.05.
- Z_β_= the Z-statistic corresponding to the power, where β = probability of type II error; usually, β is set to 1- β = 0.90 or 0.80. For our purposes, we will use 0.80.
- σ^2^= standard deviation of hs-CRP in the hypothetical study population. As reported standard deviations in included studies differed in their reported standard deviations of hs-CRP will use a standard deviation of 0.5 mg/dL.
- δ^2^= the minimum effect size we wish to detect. In our case, we will use 0.1 mg/dL.

Using these values:


$$Z_{\frac{\alpha }{2}}=Z_{0.025}=∼1.96$$

$$Z_{\beta }=Z_{0.8}=∼0.84$$

We calculate:


$$n=\frac{2.{\left(1.96+0.84\right)}^{2}.0.5^{2}}{0.1^{2}}$$

n=392

If we estimate a dropout rate of 10% or 15%, the estimated effect size increases to 436 (392/0.9) or 461 (392/0.85), respectively, per arm. Therefore, the entire study, considering a 1:1 allocation to intervention and control diet, in this scenario it is estimated that the RCT would require a sample size of between 872 (436 x 2) and 922 (361 x 2).

## CRediT author statement:

AMRH: Conceptualization, Methodology, Formal analysis, Investigation, Writing – Original Draft, Writing – Review & Editing, Visualization. AMEH: Investigation, Writing – Review & Editing. SM: Conceptualization, Methodology, Resources, Writing – Review & Editing, Supervision.
